# Palmitate enhances MSC immunomodulation of human macrophages via the ceramide/CCL2 axis in vitro

**DOI:** 10.1186/s13287-025-04536-7

**Published:** 2025-08-06

**Authors:** Courteney Tunstead, Laura M. Bitterlich, James A. Ankrum, Andrew E. Hogan, Karen English

**Affiliations:** 1 Kathleen Lonsdale Institute for Human Health Research, Maynooth University, Maynooth, Co. Kildare, Ireland; 2 Department of Biology, Maynooth University, Maynooth, Co. Kildare, Ireland; 3 University of Iowa Fraternal Order of Eagles Diabetes Research Center, University of Iowa, Iowa City, IA 52242 USA; 4 Roy J. Carver Department of Biomedical Engineering, University of Iowa, Iowa City, IA 52242 USA

**Keywords:** Mesenchymal stromal cells, Immunomodulation, Macrophages, Palmitate, Obesity, Ceramide, CCL2

## Abstract

**Background:**

The immunomodulatory function of human mesenchymal stromal cells (MSCs) strongly depends on external factors; such as cytokines and other signalling molecules encountered in the disease microenvironment. An insufficiently inflammatory environment can fail to activate MSCs, and certain signals can impair their function. Obesity is on the rise worldwide, making it an additional factor to be considered prior to MSC therapy, as the microenvironment presents its own challenges. Elevated levels of serum free fatty acids, specifically palmitate, have the potential to affect MSC therapy. Palmitate-exposure has been shown to impair MSC immunomodulation of T cells in vitro. However, this is yet to be studied in the context of macrophages.

**Methods:**

MSCs from three independent donors were exposed to 0.4mM of palmitate for 6–24 h. Gene expression, protein production and functional capacity were then assessed in response to palmitate. A ceramide synthesis inhibitor (Fumonisin B1) and a CC-chemokine ligand 2 (CCL2)-neutralising antibody were further used to assess the impact of these components on palmitate-associated immunomodulation.

**Results:**

We demonstrated that palmitate-exposed MSCs have enhanced suppression of human monocyte-derived macrophage (MDM) production of tumour necrosis factor α (TNFα), in a CCL2-dependent manner. We further elucidated parts of the pathway, such as ceramide synthesis, through which palmitate promotes this enhanced immunomodulation of macrophages.

**Conclusion:**

Palmitate-exposed MSCs show enhanced immunomodulation of human MDMs, through the ceramide/CCL2 axis in vitro.

**Supplementary Information:**

The online version contains supplementary material available at 10.1186/s13287-025-04536-7.

## Graphical Abstract

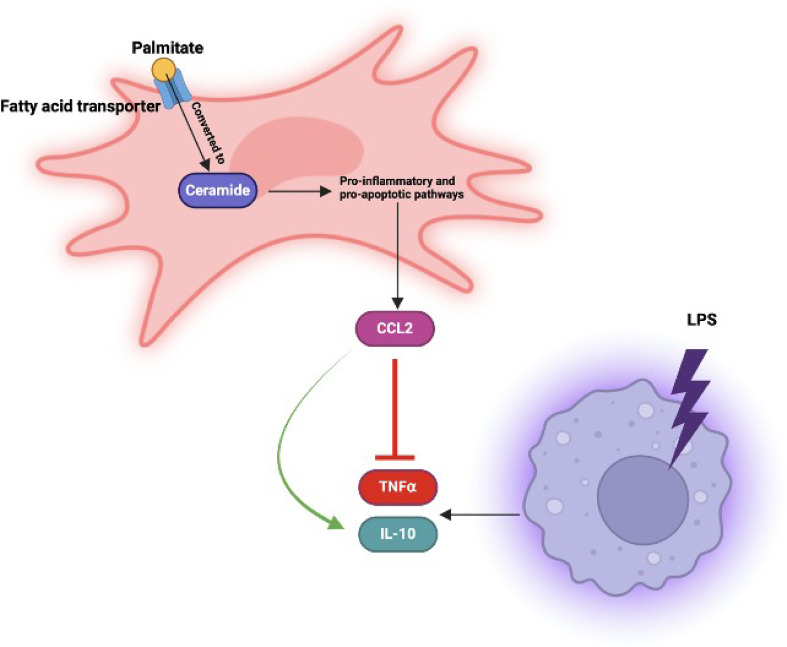

## Background

The immune calming properties of mesenchymal stromal cells (MSCs) makes them a promising therapeutic for a range of inflammatory conditions [1–5]. MSCs can suppress T cell proliferation [6] and reduce pro-inflammatory macrophage activation and function [7, 8]. Moreover, MSCs can polarise macrophages towards a more pro-resolving, non-classical, M2 phenotype [9]. In fact, the communication between MSCs and macrophages in vivo is now thought to play an essential role in MSC therapeutic efficacy; as depleting macrophages prevents MSCs from mediating their therapeutic effects [10, 11].

The microenvironment MSCs encounter upon administration to patients has an important impact of the efficacy of MSCs. MSCs require a minimal threshold of pro-inflammatory activation to carry out their immunomodulatory functions and in some cases the disease microenvironment may not provide adequate signals for this activation [14–18]. In the context of MSC administration in acute graft versus host disease (aGvHD) and in Crohn’s Fistula there is evidence that differences in patients are associated with response or non-response to MSC therapy [19, 20]. Thus, the microenvironment within patients who are to receive MSC therapy requires further investigation.

Worldwide, the number of individuals living with obesity is on the rise, with over half of the adults in the EU being overweight [21–23]. This would suggest an increase in the number of patients receiving cell-based therapies, including MSC therapy, that will also be living with the complication of obesity. Patients who are living with obesity, alongside additional inflammatory conditions, have an increased level of complexity within their disease microenvironment. In addition to increased levels of pro-inflammatory cytokines [22–24] and adipokines [25, 26], obesity is associated with elevated levels of serum free fatty acids (FFAs). Palmitate, the most abundant inflammatory FFA in the body, has been shown to exacerbate obesity-related insulin resistance through increased ceramide synthesis and inhibition of Akt phosphorylation [27, 28]. The anti-tumour response is also impacted by palmitate via de-sensitisation of monocytes and macrophages to stimulator of interferon genes (STING)-induced type-I interferon signalling, and induction of programmed cell death protein 1 (PD-1) [29, 30]. Moreover, palmitate has shown to induce endoplasmic reticulum (ER) stress in lung epithelial cells leading to apoptosis [31]. Palmitate-induced ER stress has also been described in MSCs [32], and exposure to palmitate for 48 h or more is associated with significant cell lipotoxicity [33]. Additionally, the ability of MSCs to suppress T cell proliferation in vitro is drastically impaired by the presence of palmitate, owing at least in part to decreased indoleamine 2,3-dioxygenase (IDO) activity, resulting in a lower conversion of tryptophan into kynurenine [6, 10, 34–41]. Although suppression of T cell proliferation is considered an important mechanism of action for MSCs [42, 43], recent evidence increasingly points towards an important role for MSC-macrophage interactions [19, 20, 44–46].

The aim of this study was to investigate how MSC immunomodulation of human monocyte-derived macrophages (MDMs) would be affected by exposure to palmitate in vitro. We demonstrated that palmitate significantly enhanced MSC suppression of pro-inflammatory macrophages. We identified enhanced MSC expression of *PTGS2*,* IL-6*,* CCL2* and *ANGPTL4* following exposure to palmitate. We also identified CC-chemokine ligand 2 (CCL2) as the protein responsible for mediating this improvement in MSC immunomodulation. We further elucidated the pathway through which palmitate promoted increased production of CCL2 by MSCs, by-way-of investigating ceramide de novo synthesis. We showed that palmitate led to the induction of ceramide de novo synthesis, and blockade of this pathway prevented both CCL2 production by MSCs and the associated MDM suppression.

## Methods

### Ethical approval

Ethical approval was granted by the Medical Research Ethics Committees at St Vincent’s University Hospital and by Maynooth University Ethics Committee entitled: Metabolic and Immunological Links Between Obesity, Systemic Inflammation, Type 2 Diabetes Mellitus and Non-Alcoholic Fatty Liver Disease granted on 28th June 2024 (BSRESC-2024-38575) and Investigating the role of macrophage education by MSCs in mediating MSC therapeutic efficacy granted on 11th February 2022 (BSRESC-2022-2460651). All patients gave written informed consent prior to partaking in the study.

### Human MSC culture

Human bone marrow-derived MSCs (three independent donors) were purchased from RoosterBio (Frederick, MD, USA). Initially, MSCs were expanded in RoosterBio expansion medium (RoosterBasal and RoosterBooster) for passages 1 and 2 according to the manufacturer’s instructions. After, MSCs were cultured and maintained up to passage 6 in low glucose Dulbecco’s modified Eagle medium (DMEM; Sigma-Aldrich, Wicklow, Ireland) supplemented with 10% (v/v) fetal bovine serum (FBS; ThermoFisher Scientific, Dublin, Ireland) and 1% (v/v/) penicillin/streptomycin (Sigma-Aldrich, Wicklow, Ireland). MSCs were seeded at 1 × 10^6^ cells per T175 flask and cultured at 37 °C in 5% CO_2_. Medium was replenished every 2–3 days and cells were passaged at 80% confluency. All experiments were carried out between passages 3–6. For palmitate and C2 ceramide experiments, MSCs were exposed to 0.4 mM palmitate-BSA (palmitate; Cayman Chemicals, MI, USA) or BSA as a control (6 to 24 h), 40 µM fumonisin B1 (ThermoFisher Scientific, Dublin, Ireland), or 10 µM C2 ceramide (Sigma-Aldrich, Wicklow, Ireland) or vehicle control (ethanol) (3 to 6hrs). For serum studies, MSCs (3 independent donors) were exposed to 20% of lean or obese patient serum for 24 h. This was then removed, the cells were washed with PBS, and serum-free media was added for a further 24 h. This was then harvested and CCL2 secretion was quantified by ELISA.

### Culture of human monocyte derived macrophages (MDMs)

Human peripheral blood mononuclear cells (PBMCs) were isolated from buffy coats received from the Irish Blood Transfusion Service (Saint James’ hospital, Dublin, Ireland) by lymphoprep (StemCell, Vancouver, Canada) density gradient centrifugation. PBMCs were seeded at a density of 2 × 10^6^ cells per well in tissue culture 24-well plates and allowed to adhere for 60 min. Cells were washed with Dulbecco’s Phosphate-Buffered Saline (DPBS; Merck, Cork, Ireland) to remove any non-adherent cells and medium was replaced with 300 µL cRPMI, supplemented with 5% human male AB plasma (Merck, Cork, Ireland) and topped up to 600 µL after 24 h. Monocytes were differentiated into monocyte-derived macrophages for 6 days. After 5 days, cells were washed with DPBS and medium replenished. On day 6, cells were detached by first washing them with DPBS, then adding 300 µL per well of lidocaine detachment buffer (0.5% bovine serum albumin (BSA; Merck) and 5 mg/mL lidocaine HCL (Fluorochem, Cork, Ireland)) for 20 min at 37 °C. Cells were gently pipetted up and down and transferred to a centrifugation tube. 400 µL DPBS was added to wells, remaining cells were gently dislodged using the tip of a Pasteur pipette, collected, and centrifuged at 300 g for 5 min. MDMs were then seeded into 96 well flat bottom plates at a density of 2 × 10^4^ cells per well for macrophage suppression assays.

### Flow cytometry for MDM characterisation

MDMs were detached using a lidocaine detachment buffer (0.5% BSA, 5 mg/mL lidocaine in DPBS). 2% rat serum was used to block non-specific binding of antibodies. Cells were incubated for 15 min at 4 °C with fluorescent antibodies. Cells were then first washed, then resuspended in cold flow cytometry staining (FACS) buffer (2% FBS in Dulbecco′s Phosphate Buffered Saline (DPBS/PBS)) and then acquired using the Attune Nxt flow cytometer (ThermoFisher Scientific, Dublin, Ireland). Gating was performed on live (live/dead stain, near-IR fluorescent reactive dye, Invitrogen), CD14+ (PE) cells (Supplementary Fig. 1) using antibodies against CD206 (Pacific Blue), HLA-DR (FITC), CD11b (PE-Cy7), CD86 (APC), and CD163 (PerCP). Data were analysed using floreada.io.

### Intracellular staining of COX-2

MSCs were seeded at a density of 1 × 10^5^ cells per well in tissue culture 6-well plates and allowed to adhere overnight. MSCs were then exposed to 0.4 mM palmitate-BSA (palmitate; Cayman Chemicals, MI, USA) for 24 h. After 20 h, a protein transport inhibitor cocktail containing Brefeldin A and Monensin (Invitrogen, Massachusetts, US) was added to block protein transport. Cell viability was determined using the Zombie Aqua™ Fixable Viability Kit (Biolegend, CA, USA). Cells were then washed and prepared for intracellular staining using the Foxp3/Transcription Factor Staining Buffer Set (Biosciences, Dublin, Ireland) following the manufacturer’s instructions. Samples were stained for COX-2 (PE) for 45 min. Cells were then washed in flow cytometry staining buffer (2% FBS in DPBS) and acquired using the Attune Nxt flow cytometer (ThermoFisher Scientific, Dublin, Ireland). Gating for COX-2 was performed on live single cells. Data were analysed using floreada.io.

### Enzyme-linked immunosorbent assay (ELISA)

Levels of human ANGPTL4, IL-10, TNFα, and CCL2 (R&D and BioLegend, CA, USA) in cell culture supernatant were determined using ELISA kits following the manufacturer’s instructions. Samples were diluted as necessary to stay within the range of the kits. Analysis was carried out in Corning 96-well half-area plates and volumes adjusted accordingly (ThermoFisher Scientific, Dublin, Ireland).

### Analysis of gene expression

Total ribonucleic acid (RNA) was extracted from MSCs using TRIzol (Ambion Life Sciences, Cambridgeshire, UK) following the manufacturer’s instructions. RNA concentrations were measured via spectrophotometry (Nanodrop 2000, ThermoFisher Scientific, DE, USA). For coding deoxyribonucleic acid (cDNA) synthesis, 500 ng RNA were used following the manufacturer’s instructions (Quantabio, MA, USA). Real-time polymerase chain reaction (PCR) was carried out using PerfeCta SYBR Green FastMix (Quantabio, MA, USA). Expression of genes of interest (for primer sequence information see Table 1) was qualified in relation to the housekeeping gene hypoxanthine-guanine phosphoribosyl transferase (hprt), using the ΔCT method. The fold change in gene expression relative to the control was determined via calculating the 2^−ΔΔCT^ values.

**Table 1:** Sequences for primers used in real-time PCR

Primer	Forward primer sequence (5’-3’)	Reverse primer sequence (3’-5’)
*HPRT*	ATAAGCCAGACTTTGTTGG	ATAGGACTCCAGATGTTTCC
*CERS4*	ATCCTCTACACCACATACTAC	TACGAATGTCCTTCTCCATC
*CERS5*	CTGGCATAACTATCCATTTCAG	GACCAATAGAAGGCCAATTC
*CERS6*	CTTTACATGTGTCCAAGGATG	TTGGGACTTGTAGTTTTGAG
*PTGS2*	AAGCAGGCTAATACTGATAGG	TGTTGAAAAGTAGTTCTGGG
*IL-6*	GCAGAAAAAGGCAAAGAATC	CTACATTTGCCGAAGAGC
*IDO*	TTGTTCTCATTTCGTGATGG	TACTTTGATTGCAGAAGCAG
*CCL2*	AGACTAACCCAGAAACATCC	ATTGATTGCATCTGGCTG
*ANGPTL4*	AGGCAGAGTGGACTATTTG	CCTCCATCTGAGGTCATC
*VEGFA*	AATGTGAATGCAGACCAAAG	GACTTATACCGGGATTTCTTG

### Macrophage suppression assay

MDMs were cultured as described and co-cultured with MSCs at a MSC to macrophage ratio of 1:20. MSCs were incubated with 0.4 mM palmitate-BSA (palmitate; Cayman Chemicals, MI, USA), 5 µg/mL neutralising CCL2/MCP-1 neutralising antibody (R&D Systems, Abingdon, UK), 40 µM fumonisin B1 (ThermoFisher Scientific, Dublin, Ireland), or 10 µM C2 ceramide (Sigma-Aldrich, Wicklow, Ireland) for 24 h prior to co-culture. MSCs were washed with DPBS before addition of MDMs. Co-culture was stimulated with 100 ng/mL lipopolysaccharide for 24 h (LPS; *E. coli* O111:B4, Sigma-Aldrich, Wicklow, Ireland). Supernatants were collected, and TNFα and IL-10 concentration was quantified using ELISA.

### Statistical analysis

An ordinary One-Way ANOVA with Tukey’s multiple comparisons test was performed to test for statistical significance between multiple experimental groups, and an unpaired t test with Welch’s correction was performed to test for statistical significance between two experimental groups. GraphPad Prism version 10.2.3 was used for statistical computations and graphing.

## Results

### Palmitate-enhanced Immunomodulation of MDMs by MSCs is linked to CCL2

**Fig. 1:**
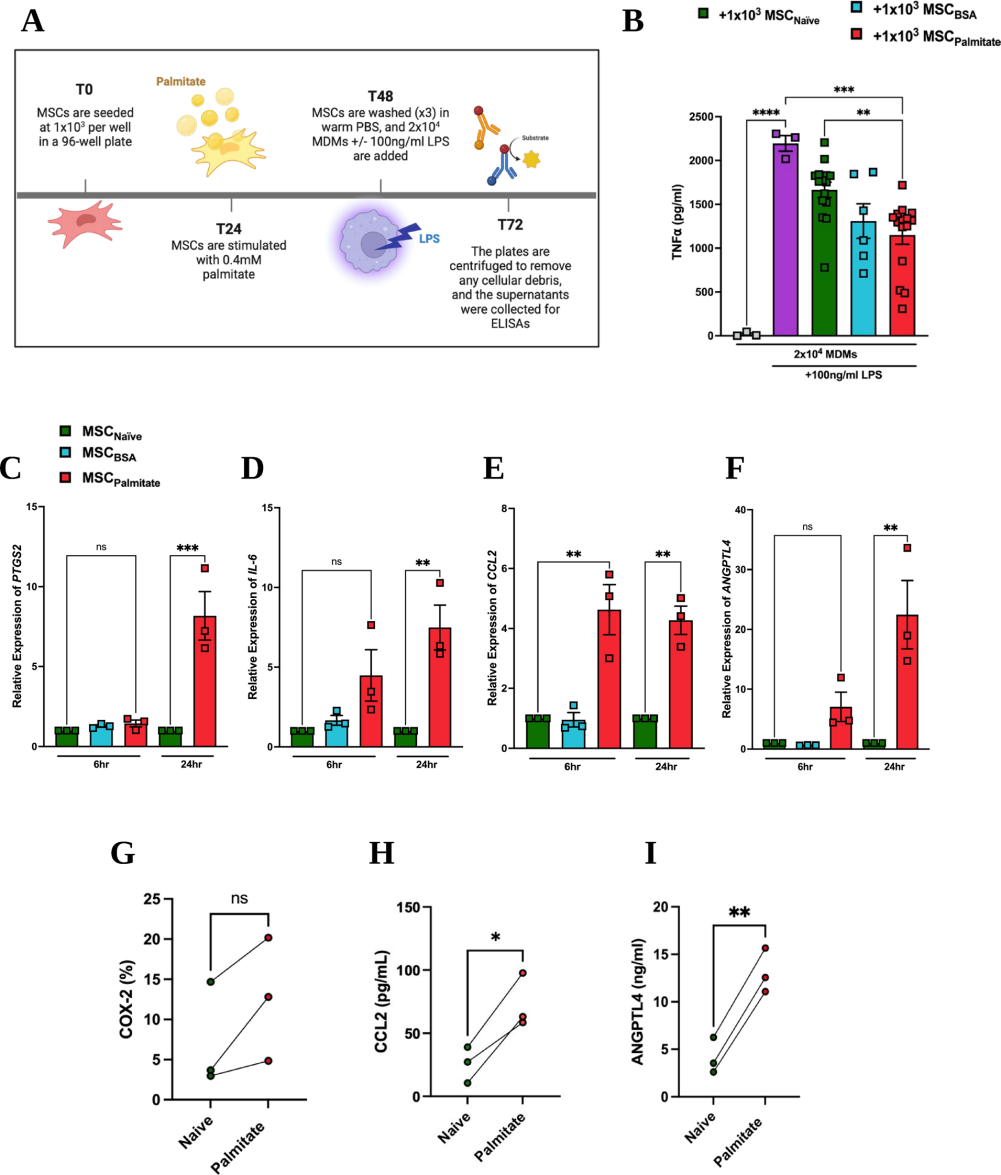
Palmitate enhances MSC immunomodulation of MDMs. (**A**) Experimental design graphic: Human bone marrow MSCs were exposed to 0.4 mM palmitate for 24 h, washed with PBS and co-cultured with human MDMs at 1:20 ratio of MSC: MDMs. The co-culture was stimulated with 100 ng/mL of LPS for 24 h, and MDM production of TNFα was measured by (**B**) ELISA (*n* = 3 MSC donors + 3–4 MDM donors). Relative gene expression of MSCs in response to 0.4 mM palmitate after 6–24 h was measured for (**C**) *PTGS2*, (**D**) *IL-6*, (**E**) *CCL2*, and (**F**) *ANGPTL4* (*n* = 3). The protein production of (**G**) COX-2, (**H**) CCL2 and (**I**) ANGPTL4 were also measured using flow cytometry (COX-2) or ELISA (CCL2 and ANGPTL4). Data is presented as mean ± SEM. *p < 0.05, **p < 0.01, ***p < 0.001, ****p < 0.0001, ns: not significant. Statistical test: Ordinary one-way ANOVA with Tukey’s multiple comparisons test (**B**-**F**) and unpaired t test with Welch’s correction (**G**-**I**)

MSCs reduce LPS-stimulated MDM production of TNFα in a dose-dependent manner (Supplementary Fig. 2). To investigate if palmitate enhanced or reduced TNFα production by MDMs, low dose MSC (1 MSC: 20 MDMs) were exposed to palmitate for 24 h and used in a macrophage suppression assay (Fig. 1A). Pre-exposure to palmitate significantly improved MSC ability to decrease the production of TNFα by MDMs compared to naive or BSA control MSCs (Fig. 1B). Others have shown a small induction of apoptosis following exposure of MSCs to palmitate for 96 h [36], we did not observe a significant induction of apoptosis following 24 h exposure to 0.4. mM palmitate (Supplementary Fig. 3). MSCs produce a multitude of immunomodulatory factors in response to pro-inflammatory stimulation [12, 47]. In response to palmitate, the expression of *PTGS2* (Fig. 1C), *IL-6* (Fig. 1D), *CCL2* (Fig. 1E), and *ANGPTL4* (Fig. 1F) were increased. While *CCL2* was significantly increased at 6 h and 24 h, *PTGS2*,* IL-6* and *ANGPTL4* were only significantly upregulated at 24 h post-palmitate exposure (Fig. 1). In contrast, MSC expression of *VEGF* and *IDO* were unaffected by palmitate exposure (data not shown).

**Fig. 2:**
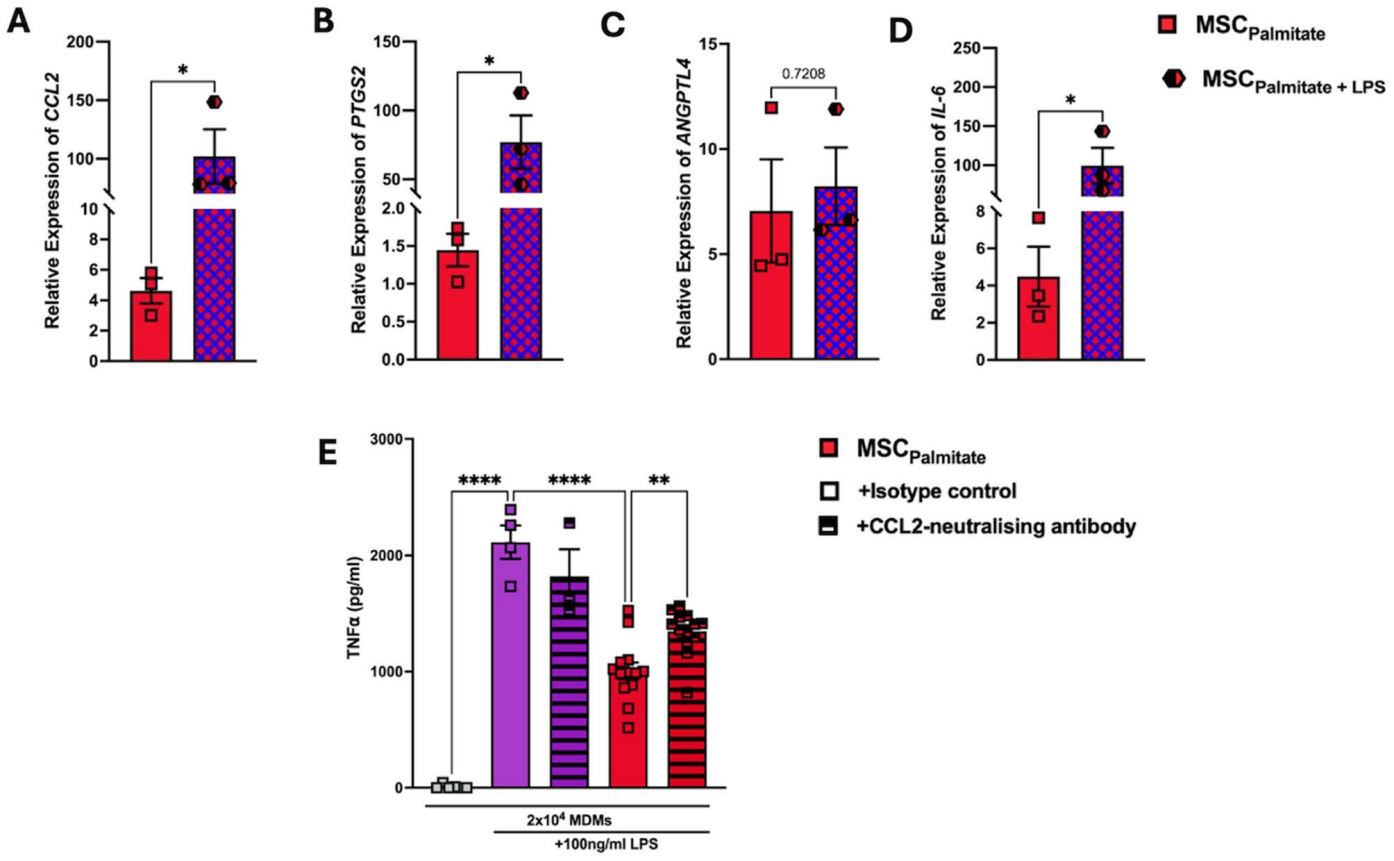
Palmitate enhances MSC immunomodulation of MDMs via increased CCL2 production. Human bone marrow MSCs that were exposed to both 0.4 mM palmitate, and 100ng/ml LPS, for 6 h and analysed for gene expression of (**A**) *CCL2*, (**B**) *PTGS2*, (**C**) *ANGPTL4* and (**D**) *IL-6* (*n* = 3). Using the same approach as seen in Fig. 1A, palmitate exposed MSCs were cocultured with MDMs (1:20 MSC: MDM ratio) and LPS (100ng/ml). A CCL2-neutralising antibody or isotype control (5 ug/mL) were added to the culture and LPS stimulated MDM production of TNFa was measured by ELISA after 24 h (**E**) (*n* = 3 MSC donors + 3–4 MDM donors). Data is presented as mean ± SEM. *p < 0.05, **p < 0.01, ****p < 0.0001. Statistical test: unpaired t test with Welch’s correction (**A**-**D**) and ordinary one-way ANOVA with Tukey’s multiple comparisons test (**E**)

Given that *PTGS2*, *CCL2* and ANGPTL4 have been associated with macrophage suppression, the gene expression results were confirmed at the protein level. While both COX-2 (Fig. 1G) CCL2 (Fig. 1H) and ANGPTL4 (Fig. 1I) protein production were increased following palmitate exposure, only CCL2 and ANGPTL4 reached significance. In our assay, naive or palmitate exposed MSCs were co-cultured with MDMs and LPS. Therefore, it was possible that changes in gene expression observed could also be mediated by LPS or the combination of palmitate and LPS. LPS and palmitate-exposed MSCs showed further enhanced expression of *CCL2*,* PTGS2* and *IL-6* but not *ANGPTL4* (Fig. 2A-D). Given the increase in both the gene and the protein expression of CCL2 (Figs. 1 and 2), we decided to pursue a CCL2-neutralisation approach. This experiment highlighted that neutralisation of CCL2 abrogated the enhanced immunosuppressive capacity of palmitate-treated MSCs (Fig. 2E).

### MSCs and palmitate stimulated MSCs promote an increase in CD206 expression by MDMs

MSCs have been shown to promote an M2 switch in LPS stimulated macrophages [48, 49]. LPS stimulated macrophages expressed significantly increased levels of the M1 activation marker CD86 and reduced levels of CD11b. Naive MSCs at a MSC: MDM ratio of 1:20 significantly increased the frequency of MDMs expressing the M2 marker CD206 but had limited effects on M1 markers CD86 or HLA-DR. Palmitate stimulated MSCs had similar effects to naive MSCs increasing the frequency of CD206 expressing cells although not significantly (Supplementary Fig. 4).

### Blocking ceramide de novo synthesis negates the effects of palmitate on MSC Immunomodulation of MDMs

**Fig. 3:**
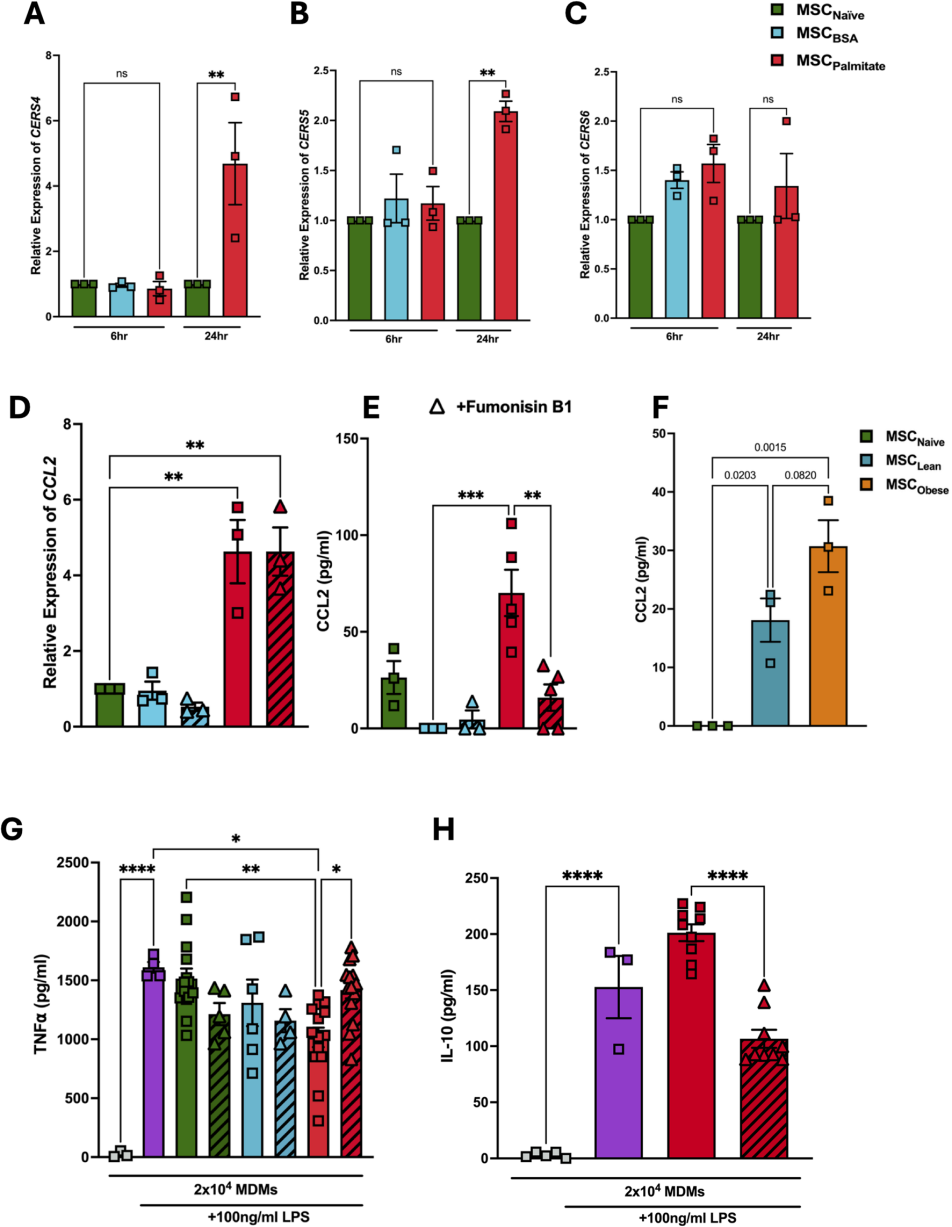
Palmitate enhances MSC immunomodulation of MDMs via ceramide de novo synthesis. Human bone marrow mesenchymal stromal cells (MSCs) were exposed to 0.4 mM palmitate for 24 h, and relative gene expression of the ceramide synthase genes (**A**) *CERS4*, (**B**) *CERS5*, and (**C**) *CERS6* was measured via qPCR (*n* = 3). MSCs were further exposed to 0.4 mM palmitate and 40 µM fumonisin B1 for 6 h (for gene expression) or 24 h (for protein production). (**D**) *CCL2* gene expression was measured by qPCR and (**E**) CCL2 production was measured by ELISA. We further confirmed CCL2 production by MSCs in response to 20% obese serum (**F**). MSCs were thoroughly washed with PBS and used in a human monocyte-derived macrophage (MDM) suppression assay at a MSCs to MDM ratio of 1:20. After 24 h of stimulation with 100 ng/mL LPS, concentration of (**G**) TNFα and (**H**) IL-10 production was measured by ELISA (*n* = 3 MSC donors + 3–4 MDM donors). It is important to note that the BSA and palmitate-treated control groups have been taken from Fig. 1B, which we added to allow for accurate comparison. Data is presented as mean ± SEM. *p < 0.05, **p < 0.01, ***p < 0.001, ****p < 0.0001. Statistical test: Ordinary one-way ANOVA with Tukey’s multiple comparisons test (**A**-**H**)

Ceramide synthases (CERS) are essential enzymes required for the de novo synthesis of ceramides [50]. Palmitate exposure significantly increased expression of the ceramide synthase (CERS) genes *CERS4* (Fig. 3A) and *CERS5* (Fig. 3B), but not *CERS6* (Fig. 3C), suggesting increased ceramide de novo synthesis in response to palmitate. Inhibition of CERS activity using fumonisin B1 [51, 52], did not affect *CCL2* gene expression at 6 h (Fig. 3D), but significantly reduced CCL2 production by MSCs in response to palmitate at 24 h (Fig. 3E). This confirmed the hypothesis that palmitate-induced production of CCL2 by MSCs was linked to the de novo synthesis of ceramide. We further confirmed CCL2 production from MSCs in response to clinically relevant samples from patients with obesity, or healthy controls (Fig. 3F). MDMs in co-culture with MSCs exposed to both palmitate and fumonisin B1 produced the same levels of TNFα as those in co-culture with BSA control MSCs (Fig. 3G, including BSA and palmitate groups seen in Fig. 1B for comparison). Interestingly, palmitate exposed MSCs enhanced IL-10 production by MDMs following LPS stimulation and addition of fumonisin B1 abrogated this effect (Fig. 3H).

### C2 ceramide can enhance MDM Immunomodulation by MSCs

**Fig. 4:**
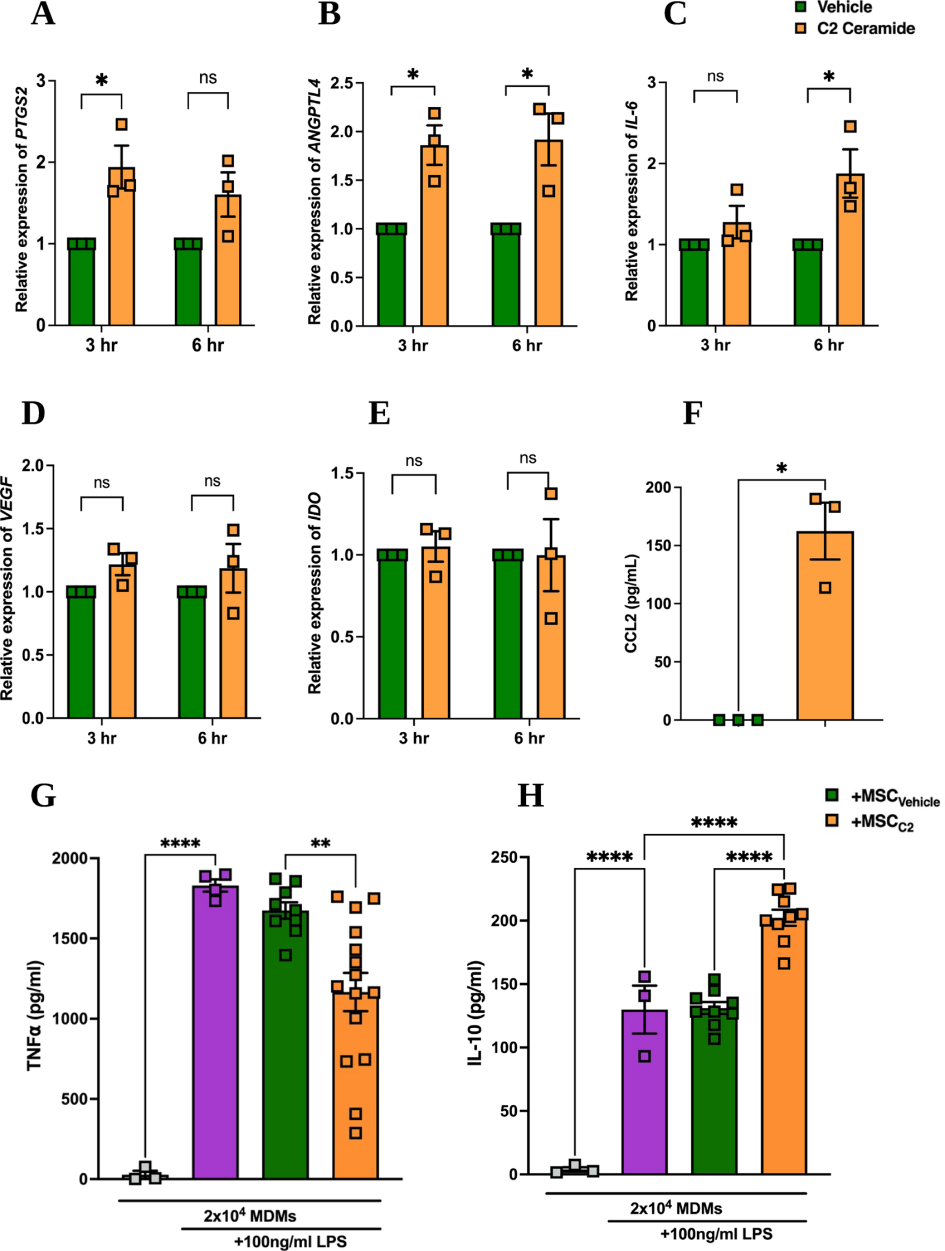
C2 ceramide exposure shows similar effects to that of palmitate in the context of MDM immunomodulation. Human bone marrow MSCs were exposed to 10 µM C2 ceramide and gene expression was measured via qPCR after 3 and 6 h for (**A**) *PTGS2*, (**B**) *ANGPTL4*, (**C**) *IL-6*, (**D**) *VEGF*, and (**E**) *IDO*. (**F**) After 24 h of exposure, CCL2 protein production was measured by ELISA (*n* = 3). MSCs were also exposed to 10 µM C2 ceramide for 24 h, thoroughly washed, and co-cultured with human MDMs at a MSCs to MDM ratio of 1:20. The co-culture was stimulated with 100 ng/mL LPS for 24 h and concentration of (**G**) TNFα and (**H**) IL-10 in the supernatant was measured by ELISA (*n* = 3 MSC donors + 3–4 MDM donors). Data is presented as mean ± SEM. *p < 0.05, **p < 0.01, ***p < 0.001, ****p < 0.0001. Statistical test: Unpaired t test with Welch’s correction (**A**-**F**) and ordinary one-way ANOVA with Tukey’s multiple comparisons test (**G**, **H**)

Aside from being used for energy generation through fatty acid oxidation, palmitate is an important substrate for the de novo synthesis of sphingolipids, specifically ceramide [53–56]. To confirm that ceramide is the crucial link between palmitate uptake and CCL2 secretion, MSCs were exposed to the cell membrane-permeable ceramide analogue: C2 ceramide [57]. Gene expression of key genes was measured after 3 and 6 h. *PTGS2* was significantly elevated at the 3 h time point, reducing at the 6 h timepoint (Fig. 4A). *ANGPTL4* was elevated at both 3 and 6 h (Fig. 4B). *IL-6* expression only increased at the 6 h time point (Fig. 4C). Neither expression of *VEGF* (Fig. 4D) nor *IDO* (Fig. 4E) were affected by exposure to C2 ceramide. Overall, these patterns in gene expression mimicked those observed in response to palmitate (Fig. 1).

MSCs were also exposed to 10 µM ceramide or vehicle control for 24 h, and CCL2 production was measured (Fig. 4F). Ceramide promoted the production of CCL2 in all three MSC donors. Ceramide-exposed MSCs also showed improved suppression of TNFα production by MDMs (Fig. 4G), and while naïve MSCs had no effect on IL-10 production, C2 ceramide pre-treated MSCs significantly increased the IL-10 production (Fig. 4H).

## Discussion

Tissue source [12, 13], donor [58–60], and recipient disease microenvironment [14, 15, 17] all influence the therapeutic efficacy of MSCs. Increasing glycolytic metabolism in MSCs by culturing them under hypoxia [42, 61, 62] or suppressing mitochondrial respiration with oligomycin [63] can drastically improve their ability to suppress the proliferation of T cells. In many cases, stimulation with pro-inflammatory cytokines like IFNγ [42, 64, 65], TNFα, and IL-1β [66–68] enhances MSC immunomodulation. Importantly there are also external factors that can impair MSC immunomodulation. Exposure to dexamethasone [69] or an activation of the proliferator-activated receptor (PPAR)-δ [70] negatively impact the ability of MSCs to suppress T cell proliferation. The same is true for MSC exposure to palmitate, with palmitate at certain concentrations even promoting a pro-inflammatory response in MSCs, leading to increased T cell proliferation [36]. Importantly, high levels of palmitate are found in the serum of patients with obesity and type 2 diabetes mellitus (T2DM) and may have a negative impact on MSC efficacy in palmitate rich environments. The interaction between MSCs and macrophages have been identified as essential in the mode of action used by MSCs to reduce or control inflammation in various inflammatory conditions [44, 71, 72]. Thus, we sought to better understand the impact that a palmitate rich environment may have on MSCs immunomodulation of MDMs in vitro.

Interestingly, palmitate did not negatively impact macrophage suppression by MSCs. Pre-exposure of MSCs to palmitate enhanced MSC suppression of macrophage-produced TNFα and led to increased IL-10 secretion in response to LPS stimulation, compared to the naive MSCs. A range of MSC secreted factors have been implicated in immunosuppression of macrophages or promotion of a more anti-inflammatory pro-resolving macrophage phenotype. Prostaglandin-endoperoxide synthase 2 (*PTGS2*), the gene encoding for COX-2, is strongly associated with MSC suppression of macrophages [8, 9, 20, 41]. MSC-derived *IL-6* plays a role in MSC homeostasis, suppression of T cell proliferation [73], and inhibition of dendritic cell differentiation [74]. *CCL2* derived from MSCs has been associated with MSC promotion of IL-10 production by macrophages [10, 44]. The enzyme IDO plays a major role in MSC suppression of T cell proliferation and MSCs have been shown to promote macrophage production of IDO [1, 6, 75, 76]. In addition to calming immune cells, MSCs can also promote angiogenesis and tissue repair via release of *ANGPTL4* [77, 78] and vascular endothelial growth factor (*VEGF*) [15].

While MSCs increased the frequency of CD206 expressing macrophages, palmitate pre-exposed MSCs did not have a greater effect on macrophage polarisation. Interestingly, palmitate pre-exposure of MSCs led to increased gene expression of *PTGS2*, *IL-6*, *CCL2*, and *ANGPTL4*, but not *VEGF* or *IDO.* Protein production of COX-2, ANGPTL4 and CCL2 was also enhanced. Boland et al. [36] have previously showed that palmitate impaired MSC suppression of T cell suppression is associated with a defect in kynurenine activity. In line with our data, Boland et al. also show enhanced expression of PTGS2 and IL-6 alongside defective kynurenine activity and loss of T cell suppression in palmitate exposed MSCs. The palmitate-induced altered signalling associated with defective T cell suppression by MSCs remains unclear. Palmitate has been shown to induce endoplasmic reticulum (ER) stress and apoptosis in MSCs [34]. For successful suppression of T cell proliferation, MSCs need to be activated by proinflammatory cytokines such as IFN-γ leading to induction of IDO production by MSCs, which then turns tryptophan into kynurenine, depriving T cells of this essential amino acid [6, 42, 75, 79, 80]. Interestingly upregulation of genes associated with lipid and sterol biosynthesis in MSCs may alter the capacity for MSCs to be activated by pro-inflammatory cytokines [81]. While COX-2 and PGE2 activity have been shown to play a partial role in MSC suppression of T cell proliferation, IDO induced kynurenine activity is thought to be the dominant mechanism. In the context of MSC suppression of macrophages COX-2, CCL2, and the phagocytosis of apoptotic MSCs have been named repeatedly as important factors [10, 38, 39, 44, 48]. Thus, our data suggest that palmitate exposure leads to enhanced production of immunomodulatory factors associated with MSC suppression of macrophages.

As neutralisation of CCL2 abrogated the effects of palmitate on MSCs in an MDM suppression assay, we concluded that CCL2 is likely the primary mechanism of action through which palmitate enhances MSC immunomodulation of MDMs. While CCL2 is primarily considered a chemoattractant, MSC-derived CCL2 has recently been associated with increased IL-10 production in macrophages and monocytes and a promotion of an M2 macrophage phenotype [10, 44, 82]. CCL2 also enhances LPS-induced IL-10 production in macrophages [83] and has been shown to promote adipose tissue macrophage infiltration [84]. Evidence from the literature shows that following i.v. administration, MSCs undergo apoptosis and release high levels of CCL2 which attract monocytes [20, 85]. Furthermore, a link between ceramide de novo synthesis from palmitate and a resulting production of CCL2 prompted by ceramide activation of the NFκB pathway has been reported in adipocytes [86, 87]. Ceramide de novo synthesis from palmitate and direct administration of ceramide have been associated with activation of nuclear factor kappa-light-chain-enhancer of activated B cells (NFκB) and p38 signalling, and subsequent production of COX-2 [88, 89]. In adipocytes, which are closely related to MSCs, palmitate exposure and de novo ceramide synthesis led to the secretion of CCL2 [86, 87, 90].

We were able to show that palmitate promotes the expression of genes associated with ceramide de novo synthesis in MSCs, and that the suppression of ceramide de novo synthesis using fumonisin B1 blocks palmitate-enhanced production of MSC derived CCL2. Fumonisin B1 also blocked the palmitate-enhanced MSC immunomodulation of MDMs, both regarding decreased TNFα and increased IL-10 production.

Finally, we were able to show that exposing MSCs directly to ceramide had similar effects to palmitate exposure, both in relation to gene expression, CCL2 production, and immunomodulation of MDMs. While palmitate likely has multiple other effects in the cell, the data suggests a role for the palmitate/ceramide/CCL2 axis in the improved MDM immunomodulation of palmitate-exposed MSCs. Although we have not identified the signalling pathways through which ceramide C2 induces CCL2 in MSCs, there is evidence from the literature showing that ceramide-enriched LDL induces CCL2 in human monocytes via activation of CD14 and TLR4 [91]. Other studies have demonstrated that palmitate enhances TLR4 signal transduction [92, 93]), and that palmitate upregulates CCL2 in pancreatic beta cells in a TLR4/MyD88/NFkB dependent manner [97]. Palmitate can also induce ER stress leading to activation of ER stress sensors IRE1a and PERK with subsequent activation of NFkB and NLRP3 signalling. Several studies have linked palmitate enhanced activation of TLR4 or TRIF/IRF3 inflammatory signalling cascades in macrophages [94–96].

While our data shows that palmitate has a beneficial effect on macrophage immunomodulation by MSCs in vitro, this finding needs to be confirmed in a more complex in vivo setting.

There are limitations of our study. The question of how palmitate induced MSC-derived CCL2 interacts with MDMs to enhance MSC immunosuppressive effects remains unanswered. We have not determined if CCL2 binds to CCR2 or another receptor on MDMs and the sequence of signalling events involved remain to be uncovered. In addition, we have not measured the effect of CCL2 neutralisation on LPS stimulated MDM production of IL-10 induced by palmitate exposed MSCs or in ceramide C2 mediated enhanced MSC suppression of MDMs.

Although an interesting finding that exposure to palmitate enhances MSC capacity to suppress cytokine production by macrophages in vitro it is unsuitable as a potential licensing strategy to enhance MSC therapeutic efficacy given the additional negative effects. However, the knowledge that a palmitate rich environment likely does not negatively affect MSC therapy in conditions where macrophages play a key role such as ARDS [97], atherosclerosis [98], and Crohn’s disease [99] may be valuable when treating patients with obesity. In fact, MSCs have been administered to T2DM patients for the treatment of diabetic nephropathy. These trials included patients with obesity (average patient BMI was defined as obese) and initial findings showed trends of stabilizing or improving eGFR and mGFR at week twelve post infusion [100, 101]. Administration of MSCs for treatment of Osteoarthritis in patients with obesity have also been shown to be efficacious [102]. A major consequence of elevated levels of palmitate in the blood is insulin resistance. Macrophages can promote insulin resistance via production of pro-inflammatory cytokines such as TNFa [103]. In preclinical models of high fat diet induced obesity there are several studies that show MSC administration improved insulin sensitivity, decreased triglyceride levels and lipotoxicity [104–107]. Indeed, MSCs have been shown to inhibit macrophage related inflammation in adipose tissue [108]. Thus, despite the negative effects of a palmitate rich environment on MSC suppression of T cell proliferation in vitro, there is a significant body of evidence to suggest that a palmitate rich environment such as that found in T2DM or obesity may not negatively impact MSC therapeutic efficacy where the mode of action involves immunomodulation of macrophages or other MSC cytoprotective functions. Combined with these published findings, our data suggests that MSCs may reduce insulin resistance via suppression of TNFa by macrophages. Moreover, our study elucidates further the role that MSC-derived CCL2 has on macrophage immunomodulation, which can be used for further research into MSC-macrophage interactions.

## Conclusion

The environment in which MSCs are exposed to will be indicative of their functional capacity in vivo. With obesity levels rising worldwide, there is an unmet need for understanding the complexities of this environment, and the impact it may have on MSC-based cell therapy. Our study, where we exposed MSCs to the highly inflammatory FFA palmitate, highlights an enhanced immunomodulatory capacity in the context of human MDMs. We further elucidated that this occurs due to the promotion of the ceramide/CCL2 axis. This study, although limited, provides novel insight into the mechanism by which palmitate-exposed MSCs aid in the immunomodulation of macrophage in vitro.

## Supplementary Information

Below is the link to the electronic supplementary material.

Supplementary Material 1: Supplementary Figure 1: Characterisation of MDM surface factors via flow cytometry. Human peripheral blood mononuclear cells (PBMCs) were isolated from buffy coats, monocytes were selected via plastic adherence and differentiated into monocyte-derived macrophages (MDMs) over 6 days. MDMs were then detached using a lidocaine detachment buffer, stained for CD14, CD86, and CD206, and analysed by flow cytometry. Some MDMs were stimulated with 100 ng/mL LPS to observe changes in CD86 expression. *n* = 3 (3 different PBMC donors)

Supplementary Material 2: Supplementary Figure 2: MSC dose dependently suppress LPS induced MDM production of TNFα. Human bone-marrow derived mesenchymal stromal cells (MSCs) and human monocyte-derived macrophages (MDMs) were co-cultured at ratios of 1:5, 1:10, and 1:20 and stimulated with 100 ng/mL LPS for 24 hr. MDM production of TNFα was measured by ELISA. *n* = 3 (3 different PBMC donors, 1 MSC donor). Data are presented as mean ± SEM. **p<0.01, ****p<0.0001. Statistical test: Ordinary one-way ANOVA with Tukey’s multiple comparisons test

Supplementary Material 3: Supplementary Figure 3: Analysis of palmitate induction of apoptosis in MSCs. MSCs were exposed to 0.4 mM or 1 mM palmitate or 0.5 uM Staurosporine as a positive control for 24 hr. MSC viability and induction of apoptosis was examined using an Annexin V/PI assay. *N*=3, 3 independent MSC donors. Data are presented as mean ± SEM.

Supplementary Material 4: Supplementary Figure 4: MSCs promote an M2 switch in MDMs, and this is not further enhanced by palmitate pre-exposed MSC. MSCs from 3 donors were seeded at 2.5x10^3^ cells/well in a 24 well plate and treated with 0.4 mM palmitate for 24 hr. MSCs were then washed thoroughly twice with warm PBS and 5x10^3^ MDMs were added in abRPMI for an MSC to MDM ratio of 1:20. The co-culture was stimulated with 100 ng/mL LPS for 24 hr and cells were harvested using a lidocaine detachment buffer. Cells were incubated with fluorochrome labelled antibodies and surface phenotype was analysed using the Attune Nxt flow cytometer. Gating was performed on live (live/dead stain, near-IR fluorescent reactive dye, Invitrogen), CD14+ (PE) cells using antibodies for CD206 (Pacific Blue), HLA-DR (FITC), CD11b (PE-Cy7), CD86 (APC), and CD163 (PerCP). Data were analysed using floreada.io. Statistical test: Ordinary one-way ANOVA with Tukey’s multiple comparisons test *p<0.05, ns; not significant. *n* = 3 human MDM donors.

## Data Availability

The datasets used and/or analysed during the current study are available from the corresponding author on reasonable request.

## Declarations

### Use of AI

The authors declare that they have not used AI-generated work in this manuscript.

## Abbreviations

ANGPTL4: Angiopoietin-like 4
APC: Allophycocyanin
BSA: Bovine serum albumin
CCL: CC-chemokine ligand
cDNA: Coding deoxyribonucleic acid
CERS: Ceramide synthase
COX-2: Cyclooxygenase-2
DMEM: Dulbecco’s modified Eagle medium
DPBS/PBS: Dulbecco′s Phosphate Buffered Saline
ELISA: Enzyme linked immunosorbent assay
FACS: Flow cytometry staining
FBS: Fetal bovine serum
FFA: Free fatty acid
FPKM: Fragments per kilobase million
HPRT: Hypoxanthine-guanine phosphoribosyl transferase
IDO: Indoleamine 2,3-dioxygenase
IFN: Interferon
IL: Interleukin
LCFA: Long-chain fatty acid
LPS: Lipopolysaccharide
NFκB: Nuclear factor kappa-light-chain-enhancer of activated B cells
Palmitate: Palmitate-BSA
PBMCs: Peripheral blood mononuclear cells
PCR: Polymerase chain reaction
PE: R-Phycoerythrin
PGE2: Prostaglandin E₂
PTGS2: Prostaglandin-endoperoxide synthase 2
RBC: Red blood cell
SCFA: Short-chain fatty acid
SLC27: Solute carrier family 27
TNF: Tumour necrosis factor
VEGF: Vascular endothelial growth factor
