# Supplementary material for: Palmitate enhances MSC immunomodulation of human macrophages via the ceramide/CCL2 axis in vitro
Source: Stem Cell Res Ther. 2025 Aug 6;16:435. doi: 10.1186/s13287-025-04536-7 (PMC12329961; doi:10.1186/s13287-025-04536-7)
Supplement: Supplementary file 1 — Supplementary Material 1: Supplementary Figure 1: Characterisation of MDM surface factors via flow cytometry. Human peripheral blood mononuclear cells (PBMCs) were isolated from buffy coats, monocytes were selected via plastic adherence and differentiated into monocyte-derived macrophages (MDMs) over 6 days. MDMs were then detached using a lidocaine detachment buffer, stained for CD14, CD86, and CD206, and analysed by flow cytometry. Some MDMs were stimulated with 100 ng/mL LPS to observe changes in CD86 expression. n = 3 (3 different PBMC donors) [file 13287_2025_4536_MOESM1_ESM.docx]

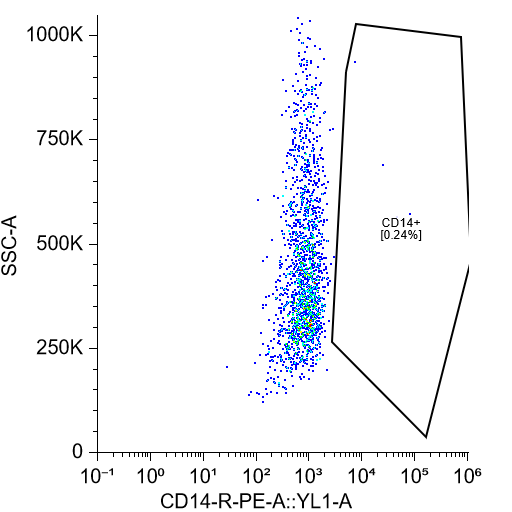

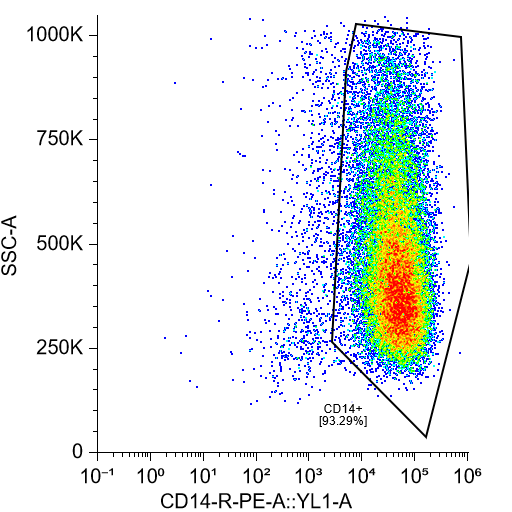

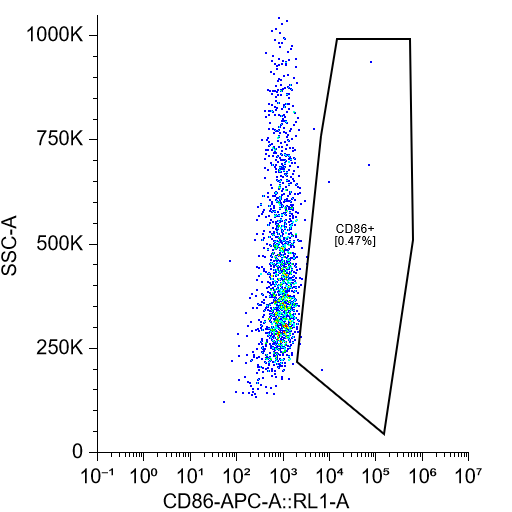

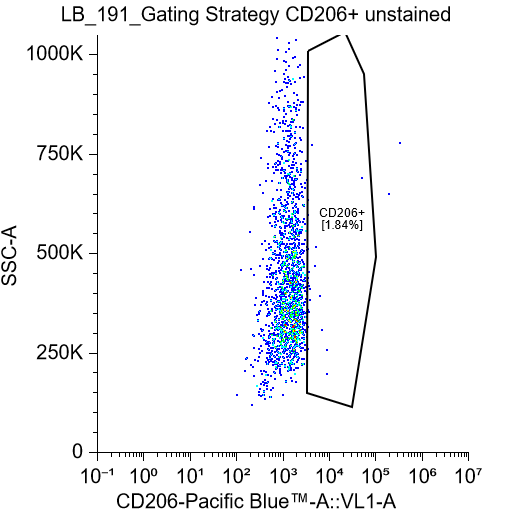

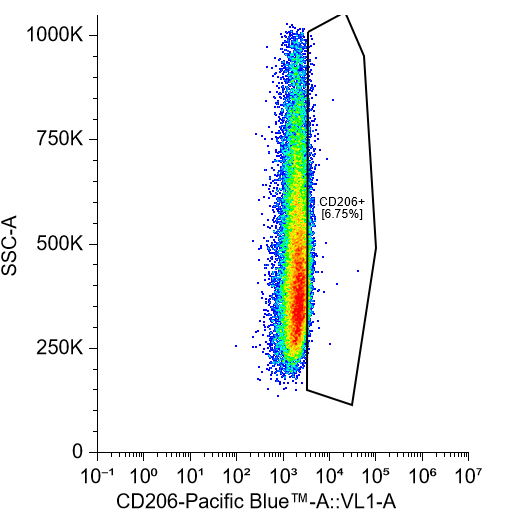

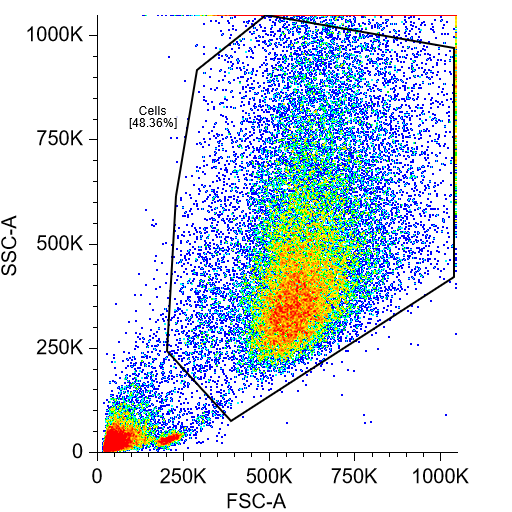

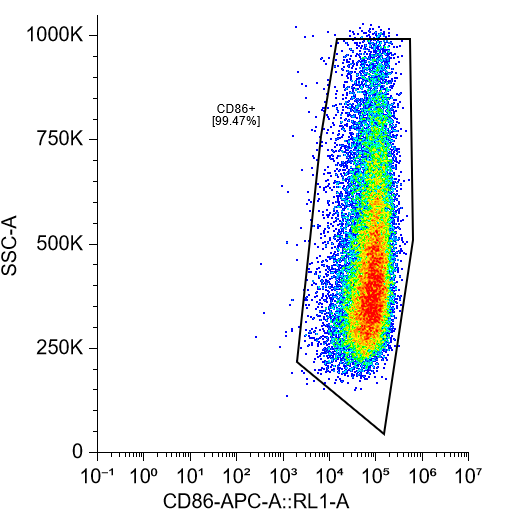

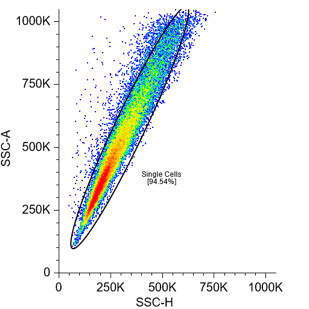

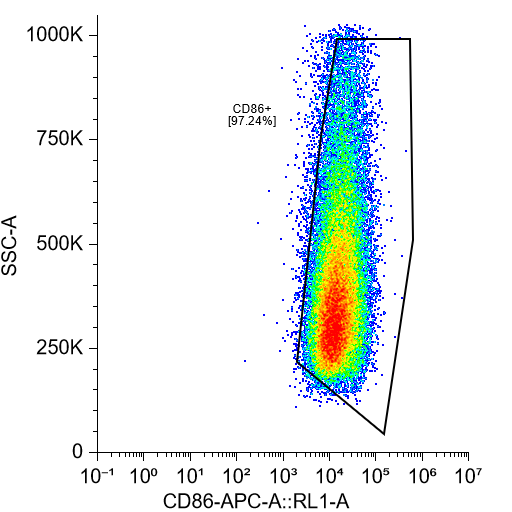


Exclusion of debris

Gating for single cells

CD14+

CD86+

CD206+

unstained

unstained

unstained

LPS stimulated

unstimulated
