# Supplementary figures and images for: Palmitate enhances MSC immunomodulation of human macrophages via the ceramide/CCL2 axis in vitro
Source: Stem Cell Res Ther. 2025 Aug 6;16:435. doi: 10.1186/s13287-025-04536-7 (PMC12329961; doi:10.1186/s13287-025-04536-7)

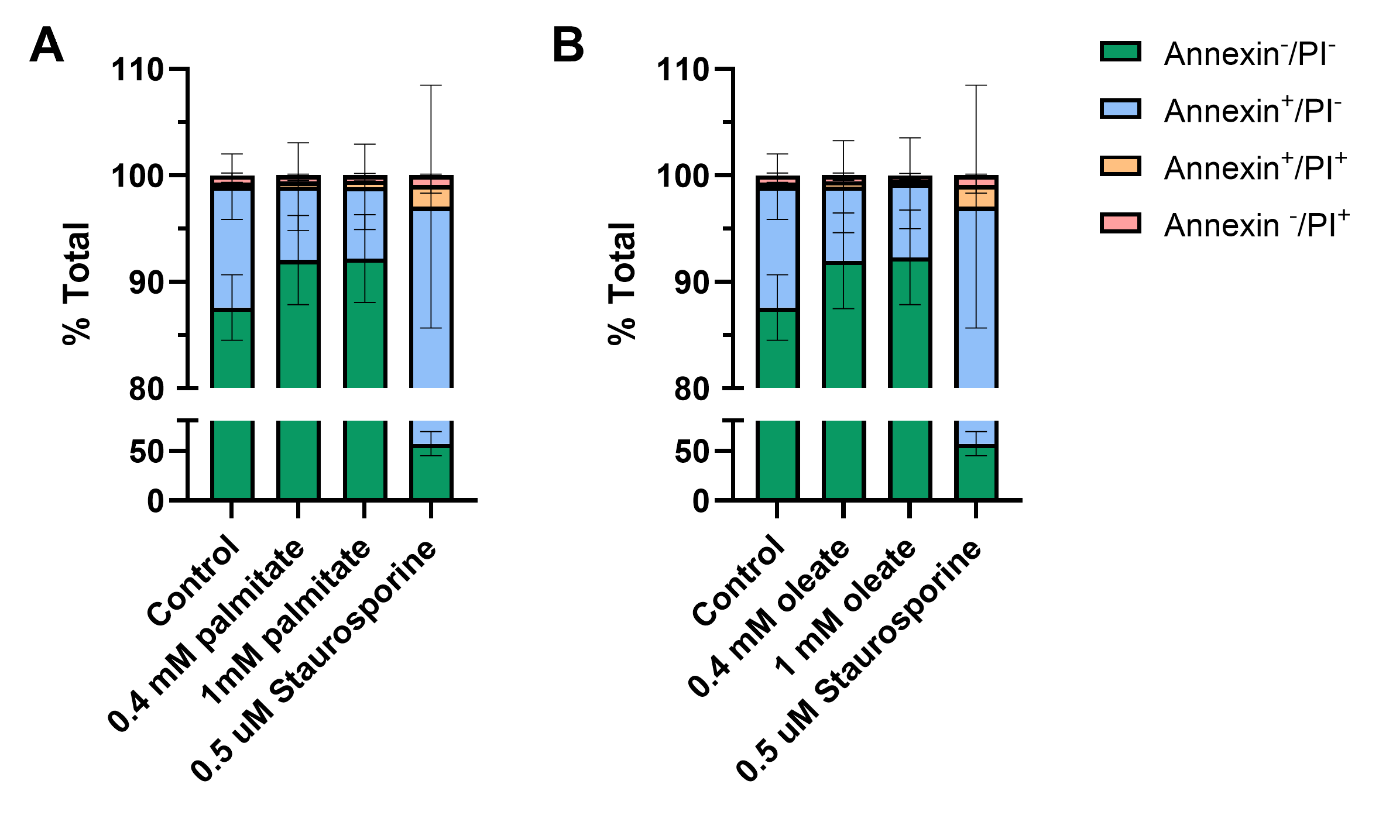

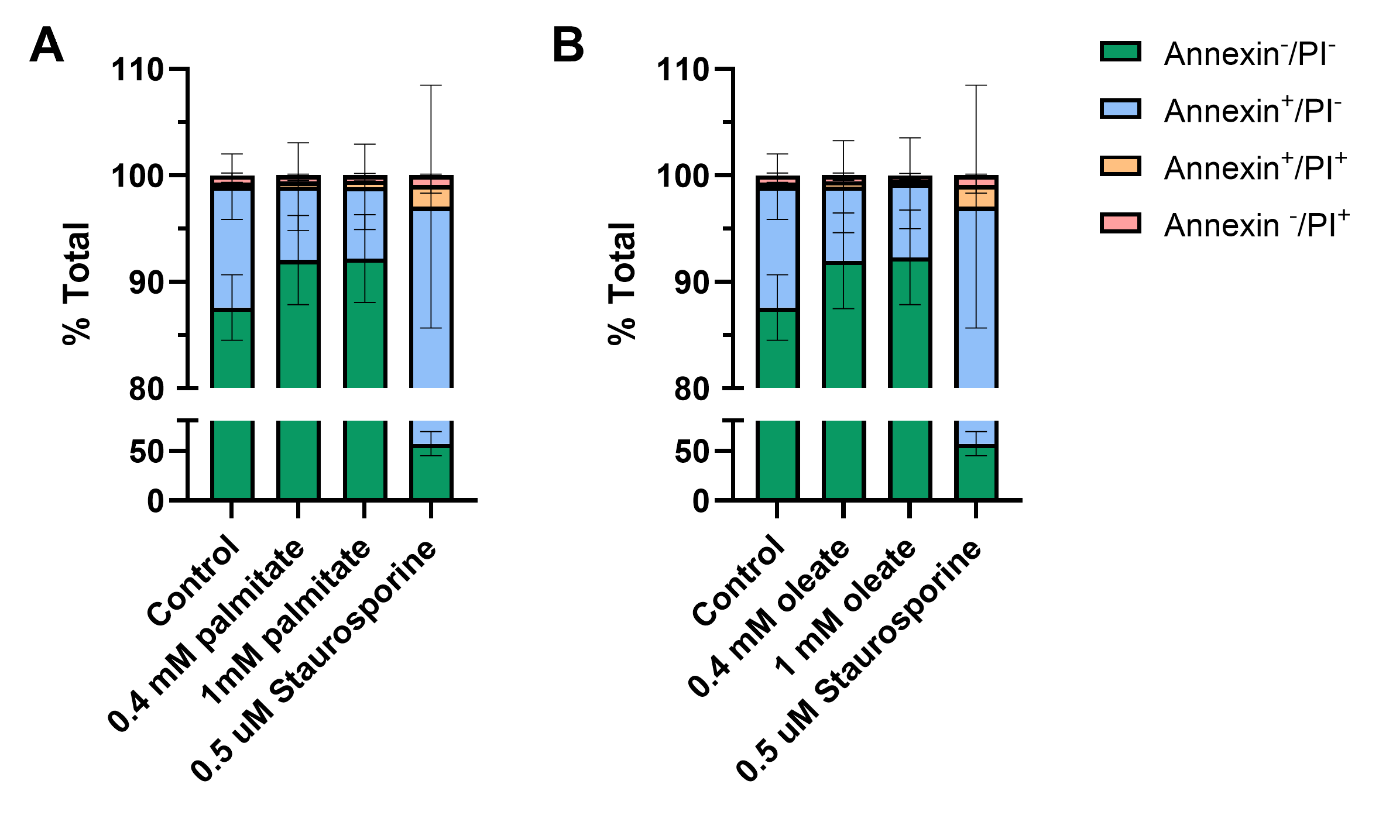

Supplement: Supplementary file 3 — Supplementary Material 3: Supplementary Figure 3: Analysis of palmitate induction of apoptosis in MSCs. MSCs were exposed to 0.4 mM or 1 mM palmitate or 0.5 uM Staurosporine as a positive control for 24 hr. MSC viability and induction of apoptosis was examined using an Annexin V/PI assay. N=3, 3 independent MSC donors. Data are presented as mean ± SEM. [file 13287_2025_4536_MOESM3_ESM.docx]
