# Supplementary material for: Palmitate enhances MSC immunomodulation of human macrophages via the ceramide/CCL2 axis in vitro
Source: Stem Cell Res Ther. 2025 Aug 6;16:435. doi: 10.1186/s13287-025-04536-7 (PMC12329961; doi:10.1186/s13287-025-04536-7)
Supplement: Supplementary file 4 — Supplementary Material 4: Supplementary Figure 4: MSCs promote an M2 switch in MDMs, and this is not further enhanced by palmitate pre-exposed MSC. MSCs from 3 donors were seeded at 2.5x10^3^ cells/well in a 24 well plate and treated with 0.4 mM palmitate for 24 hr. MSCs were then washed thoroughly twice with warm PBS and 5x10^3^ MDMs were added in abRPMI for an MSC to MDM ratio of 1:20. The co-culture was stimulated with 100 ng/mL LPS for 24 hr and cells were harvested using a lidocaine detachment buffer. Cells were incubated with fluorochrome labelled antibodies and surface phenotype was analysed using the Attune Nxt flow cytometer. Gating was performed on live (live/dead stain, near-IR fluorescent reactive dye, Invitrogen), CD14+ (PE) cells using antibodies for CD206 (Pacific Blue), HLA-DR (FITC), CD11b (PE-Cy7), CD86 (APC), and CD163 (PerCP). Data were analysed using floreada.io. Statistical test: Ordinary one-way ANOVA with Tukey’s multiple comparisons test *p<0.05, ns; not significant. n = 3 human MDM donors. [file 13287_2025_4536_MOESM4_ESM.docx]

MDMs + LPS

MDMs + LPS + MSCs (naïve)

MDMs + LPS + MSCs (palmitate)

MDMs

**A**

**B**

**C**

**D**

**E**
