# Supplementary material for: Palmitate enhances MSC immunomodulation of human macrophages via the ceramide/CCL2 axis in vitro
Source: Stem Cell Res Ther. 2025 Aug 6;16:435. doi: 10.1186/s13287-025-04536-7 (PMC12329961; doi:10.1186/s13287-025-04536-7)
Supplement: Supplementary file 4 [file 13287_2025_4536_MOESM2_ESM.docx]

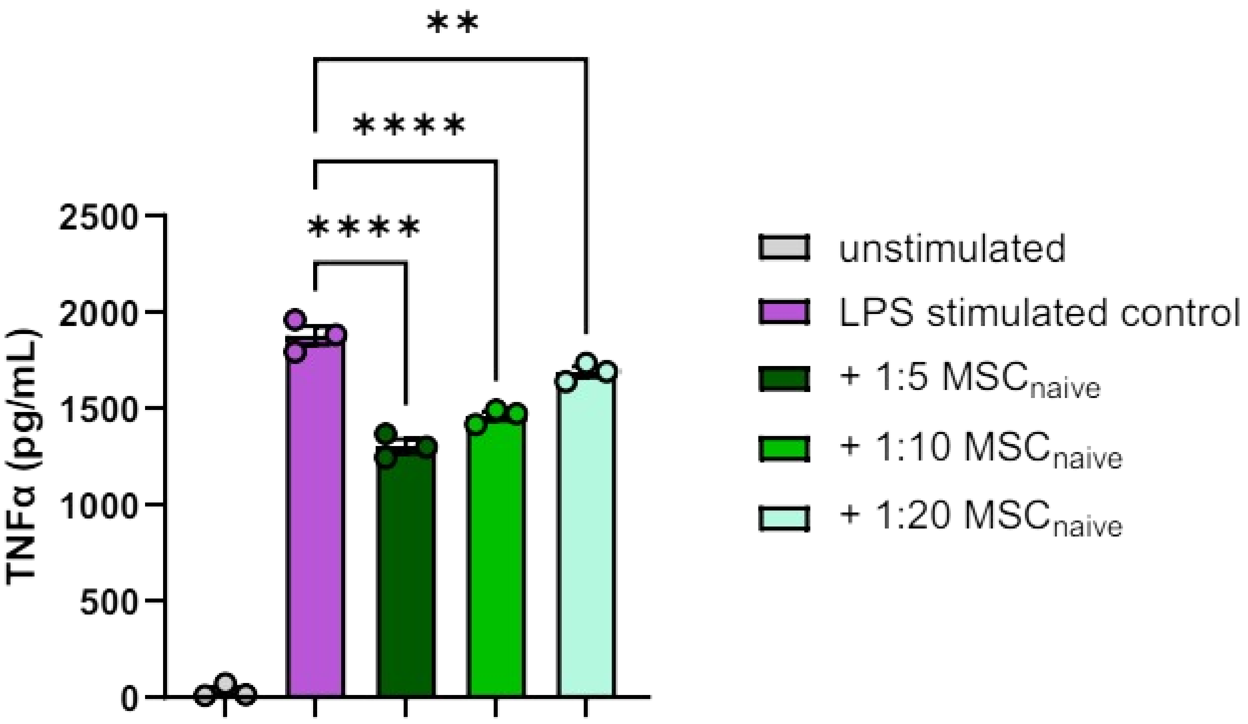

**
****
****
2500
2000
1500
1000
500
0
TNFα (pg/mL)
unstimulated
LPS stimulated control
+ 1:5 MSCnaive
+ 1:10 MSCnaive
+ 1:20 MSCnaive
